# Transcriptional regulation of Tfh dynamics and the formation of immunological synapses

**DOI:** 10.1038/s12276-024-01254-7

**Published:** 2024-06-03

**Authors:** Ye-Ji Kim, Jinyong Choi, Youn Soo Choi

**Affiliations:** 1 Department of Biomedical Sciences, Seoul National University College of Medicine, Seoul, Korea; 2 Department of Microbiology, Department of Biomedicine & Health Sciences, College of Medicine, The Catholic University of Korea, Seoul, Korea; 3 Department of Medicine, Seoul National University College of Medicine, Seoul, Korea; 4 Transplantation Research Institute, Seoul National University Hospital, Seoul, Korea

**Keywords:** Germinal centres, Immunogenetics

## Abstract

Inside germinal centers (GCs), antigen-specific B cells rely on precise interactions with immune cells and strategic localization between the dark and light zones to clonally expand, undergo affinity maturation, and differentiate into long-lived plasma cells or memory B cells. Follicular helper T (Tfh) cells, the key gatekeepers of GC-dependent humoral immunity, exhibit remarkable dynamic positioning within secondary lymphoid tissues and rely on intercellular interactions with antigen-presenting cells (APCs) during their differentiation and execution of B-cell-facilitating functions within GCs. In this review, we briefly cover the transcriptional regulation of Tfh cell differentiation and function and explore the molecular mechanisms governing Tfh cell motility, their interactions with B cells within GCs, and the impact of their dynamic behavior on humoral responses.

## Tfh cell motility and B cell interactions drive immune response

This review focuses on T follicular helper (Tfh) cells—cells that are vital to antibody (Ab) production of the immune system. Led by Y.S. Choi, the team outlines how Tfh cells collaborate with B cells in our lymph nodes (small, bean-shaped glands throughout the body) to combat infections. The authors delve into genetic and molecular processes dictating Tfh cells’ actions. Key takeaways highlight the need for precise positioning and timing of Tfh cells with B cells, as well as the critical role of Tfh cell-B cell interaction intensity in B cell affinity maturation. The authors conclude the article by emphasizing the importance of gaining insights into these interactions to develop new therapeutic strategies for autoimmune diseases (conditions where the immune system mistakenly attacks the body), and to enhance vaccine responses. This summary was initially drafted using artificial intelligence, then revised and fact-checked by the author.

## Introduction

In the intricate landscape of secondary lymphoid tissues, a remarkable choreography unfolds, orchestrated by components of the innate and adaptive immune system. This immunological program involves sophisticated interactions between innate and adaptive immune cells in an antigen (Ag)-specific manner, culminating in the generation of effector cells^1^. At the core of this intricate program lies the germinal center (GC), an immunological engine compelling Ag-specific B cells clonally expand and evolve their Ag receptors through AID (activation-induced cytidine deaminase)-mediated somatic hypermutation. This process leads to a gradual increase in the Ag-binding strength of B-cell receptors (BCRs). Within GCs, long-lived plasma cells or memory B cells emerge^2^. The complex behavior of GC B cells is meticulously regulated by the immunological functions of CD4 T cells^1,2^.

Histological findings of CD4 T cells in the GC areas of human tonsils introduced the practical concept that CD4 T cells, which aid B cells, are colocalized in follicles with B cells^3^. While this may seem obvious now, limited information was available about the specific positioning of B helper CD4 T cells, despite their existence being known for decades. Transcriptomic analyses of follicle-resident CD4 T cells in humans and mice opened Pandora’s box of a new lineage of effector CD4 T cells. These B-cell helper effector CD4 T cells, known as follicular helper CD4 T (Tfh) cells, exhibited a unique gene expression profile in comparison to those of other effector CD4 T cells, and B-cell lymphoma-6 (Bcl6) was identified as a major lineage-determining transcription factor^1,4^.

In secondary lymphoid organs, naive Ag-specific CD4 T cells develop into effectors in the T-cell zone during interaction with Ag-presenting dendritic cells (DCs). Early bifurcation between Tfh and non-Tfh cells occurs during this stage^5^, and these effector CD4 T cells exhibit differential migratory and intercellular interaction behaviors. Tfh cells stay and migrate to the B:T border, where they interact with cognate B cells to further polarize into germinal center Tfh (GC-Tfh) cells, while non-Tfh cells migrate to peripheral effector sites to fulfill immunological functions. GC-Tfh cells induce GC formation in B-cell follicles. Via recurring interactions with B cells, GC-Tfh cells play important roles in the selection of clones that express affinity-matured BCRs, the provision of signals required for B cells to undergo GC reactions, and B cell differentiation into memory B cells or long-lived plasma cells^1^.

Tfh differentiation necessitates a judicious control given the critical roles in inducing GC responses, one of the most powerful responses of the immune system. The pivotal process requires Tfh cells to distinguish cognate B cells from myriad B-cell clones with irrelevant antigen specificities in lymphoid tissues^6–8^. To guarantee this important checkpoint, a delicate balance of signaling pathways and transcription factor activity is required to control the expression of chemokine receptors and molecules regulating CD4 T-cell dynamics, such as positioning and migration, as well as the expression of costimulatory molecules involved in the interaction of CD4 T cells with B cells.

In this review, we explore the complex dynamics of Tfh cells, beginning with an exploration of the transcriptional regulation that underpins their differentiation and function. We then review the current status of our understanding of the molecular regulation of Tfh cell positioning and the intricacies of Tfh cell interactions with B cells. We will then discuss the consequences of dysregulated Tfh cell dynamics and B:T synapse formation in various physiological contexts, such as vaccination and autoimmune diseases.

## Tfh cell differentiation

Ag-specific CD4 T cells develop into pre-Tfh cells during interactions with antigen-presenting cells (APCs) in the T-cell zone, similar to other effector CD4 T cells (Th1, Th2, or Th17)^1^. Pre-Tfh cells migrate toward the boundary between the T-cell zone and B-cell follicles (B:T border), where Tfh cells interact with B cells that present cognate peptide Ags. Cognate interaction is an important checkpoint for Tfh cells to further polarize into GC-Tfh cells. This process leads to further enhancement of Tfh identity, resulting in proper localization within the B-cell follicle for interaction with GC B cells^6^.

### Positive regulators of Tfh differentiation

The Tfh differentiation pathway necessitates stable and sequential interactions with APCs, such as non-B-cell APCs in the T-cell zone and B cells at the B:T border and inside GCs. This notion implies that signaling pathways downstream of T-cell receptors (TCRs) and costimulatory molecules could create a conducive environment for Tfh differentiation. TCRs with greater affinity for peptide Ags enable prolonged interactions with DCs in the T-cell zone, enhancing Tfh differentiation^9,10^. Inducible T-cell costimulator (ICOS), which interacts with ICOS ligand (ICOS-L) expressed on DCs and B cells, acts as a positive regulator of Tfh differentiation^5^. ICOS-mediated activation signals not only induce Bcl6 and c-Maf, transcription factors that positively regulate Tfh differentiation (discussed below) but also inhibit Foxo1, Blimp1, and T-bet, transcription factors that antagonize Tfh differentiation (discussed in the following section), partly via PI3K-mediated activation of Akt and TBK1^11–13^.

The identification of Bcl6 as a fate-determining transcription factor for Tfh differentiation led to the acknowledgment of Tfh cells as a distinct lineage of effector CD4 T cells. Bcl6 centrally locates in Tfh cell development by shaping their distinct gene expression profiles and functional attributes by upregulating genes related to Tfh identity, follicular migration, and B-cell interactions^4,14–18^. In addition to Bcl6, numerous transcription factors, including Ascl2, TCF1, c-Maf, Batf, and IRF4^4,8^, also play critical roles in Tfh differentiation. Ascl2, a member of the basic helix-loop-helix (bHLH) transcription factor family, contributes to Tfh cell differentiation by collaborating with Bcl6 to enhance CXCR5 expression^19^. TCF1 cooperates with LEF1 to drive early Tfh differentiation by activating pro-Tfh genes such as *Bcl6*, *Icos*, and *Il6st* while inhibiting anti-Tfh genes such as *Il2ra* and *Prdm1*^20–23^. c-Maf, a member of the Maf family of transcription factors, and IRF4 were also found to positively regulate Tfh differentiation via IL-21 cytokine production and its signaling pathway in CD4 T cells^24–29^. Batf acts during the early stage of Tfh differentiation by actively inducing Bcl6 and Maf in CD4 T cells^27^.

Tfh differentiation is also regulated by cytokines. IL-6 induces c-Maf, which in turn activates IL-21 production in activated CD4 T cells^30^. By sharing downstream STAT1 and STAT3 transcription factors, IL-6 and IL-21 synergistically induce Tfh differentiation via transcriptional activation of target genes, including *Bcl6*^31,32^. Recently, IL-6 was revealed to inhibit the upregulation of the beta subunit of the IL-2 receptor complex (IL-2Rβ) by impeding STAT5 binding to the *Il2rb* locus, rendering Tfh and GC-Tfh cells, which are strong IL-2 producers, less responsive to autocrine IL-2-mediated inhibition of Tfh differentiation^33^. In humans, Tfh differentiation can be induced by IL-12 via STAT4 activation during in vitro stimulation^8,34^.

### Negative regulators of Tfh differentiation

Bcl6 and Blimp1 are reciprocally antagonistic to each other in the fate determination of B cells, deciding whether to undergo Bcl6-driven GC formation or Blimp1-mediated plasma cell differentiation^35–37^. In CD4 T cells, Blimp1 antagonizes Bcl6-dependent Tfh differentiation^16^. Abundantly expressed in non-Tfh cell lineages, Blimp1 restricts Bcl6 expression and downregulates genes associated with Tfh differentiation, particularly CXCR5^14,16^. Foxo1, Foxp1, Klf2, and Bach2 were also revealed to inhibit Tfh differentiation via transcriptional induction of *Prdm1*, a gene encoding Blimp1, or direct inhibition of pro-Tfh genes (*Il21*, *Cxcr5*, and *Bcl6*)^24,38–41^.

While IL-2 was originally identified as a T-cell growth factor^42^, recent studies revealed its important role in inhibiting Tfh differentiation^9,43,44^. Robust IL-2 signaling due to enhanced expression of IL-2Rα, the high-affinity IL-2 receptor, or high IL-2 bioavailability leads to strong phosphorylation of STAT5, which represses Bcl6 via Blimp1 induction^16,44^. Anti-IL-2 monoclonal antibodies enhanced Tfh differentiation of murine CD4 T cells following exogenous stimulation and maximal Tfh differentiation during in vitro stimulation of human CD4 T cells, highlighting the physiological relevance of IL-2-dependent inhibition of Tfh differentiation^44,45^. By sharing the common gamma chain (γc) with IL-2, IL-7 also negatively regulates Tfh differentiation. It functions in a slightly different manner from IL-2. STAT5 activated by IL-7 competes with STAT3 and inhibits STAT3-mediated Bcl6 induction rather than activating Blimp1 expression^46^.

### Interplay of transcription factors in Tfh differentiation

The dynamic balance between positive and negative regulators ensures the stage-specific control of Tfh differentiation, the maintenance of Tfh integrity, and the proper localization of Tfh cells. A recent study revealed that Bcl6 is central to this regulatory network. As a nexus transcriptional repressor, Bcl6 orchestrates the Tfh differentiation program by (1) directly repressing transcription factors such as T-bet, GATA-3, RORγt, STAT5, and Blimp1, which are crucial inducers of alternative helper T-cell lineages, and (2) inducing molecules essential for Tfh differentiation (i.e., CXCR5, SAP, PD-1, ICOS, and CD40L) through a repression-of-repressor mechanism^4,14^. Bcl6 inhibits Blimp1, Id2, and Runx3 from repressing CXCR5 expression, whereas Klf2-mediated suppression of PD-1 and S1PR1 is released via direct repression of Klf2 by Bcl6^4^.

### Epigenetic regulation of Tfh differentiation

Epigenetic studies have ushered in a new era in our efforts to understand how CD4 T cells control the development of effector CD4 T cells. In the context of the Tfh differentiation program, epigenetic regulators have been shown to collaborate with transcription factors or act independently to control genes involved in the Tfh differentiation pathway. For instance, Ten-eleven translocation 2 (Tet2), one of the three Tet methylcytosine dioxygenases, negatively regulates Tfh differentiation^47^. Tet2 deletion in CD4 T cells hence leads to enhanced differentiation of CD4 T cells into GC-Tfh cells. Mechanistically, Tet2 coordinates with Foxo1 to mediate demethylation at the *Runx2 and Runx3* loci, inducing the expression of these genes^47^. Other chromatin regulators also impact Tfh differentiation. Ezh2 histone methyltransferase is recruited by TCF1 to activate Bcl6 transcription and facilitate Tfh differentiation^48^. The histone methyltransferase mixed lineage leukemia 1 (Mll1) also functions as a positive regulator of Tfh differentiation. Deletion of *Mll1* in CD4 T cells led to significant reductions in Bcl6, LEF-1 and TCF-1 expression and consequently resulted in impaired Tfh differentiation of Ag-specific CD4 T cells during acute viral infections or protein immunizations^49^. Given the spatiotemporal nature inherent to Tfh differentiation and function, which are orchestrated by a series of transcription factors, it is imperative to elucidate how epigenetic modifications of Tfh-inducing or -antagonizing transcription factors may modulate the target genes governing Tfh differentiation.

## Dynamics of Tfh cells

To elicit T cell-dependent (TD) humoral immunity from Ag-specific B cells, Tfh cells need to meet at least two conditions: colocalization with B cells and GC B cells and provision of immunological nutrients. Inside GCs, GC B cells repeatedly migrate between the dark and light zones, where Ag-specific B cells undergo somatic hypermutation during proliferation and are clonally selected by GC-Tfh cells via BCR affinity-dependent competition^2,50^. GC-Tfh cells employ chemokine receptors and B-cell-interacting molecules to properly localize in the light zone and to ensure that GC B cells that have successfully acquired affinity maturation for Ags are selected^2^.

### Cellular dynamics of GCs

GC-Tfh cells, GC B cells, and follicular dendritic cells (FDCs) actively interact inside GCs^51^. After undergoing cell division and somatic hypermutation in the dark zone, GC B cells migrate back to the light zone. In the light zone, GC B cells with diverse BCR sequences compete for Ags captured by FDCs, and cells with higher Ag affinity BCRs can take up and process Ags, presenting peptide Ags on MHC II molecules. GC B cells expressing cognate peptide Ags interact with GC-Tfh cells, receive signals to migrate back to the dark zone and undergo another round of the cell division-somatic hypermutation-positive selection cycle^7,51,52^.

The dynamic migration of GC B cells is controlled by chemokine receptors. Sphingosine-1-phosphate receptor 2 (S1PR2), coupled with the heterotrimeric G protein Gα13, is important for maintaining GC B-cell localization inside GCs. A deficiency in the *Gα13* gene led to rapid dissemination of GC B cells into circulation^53^. The cyclic movement of GC B cells between the dark and light zones is controlled by the modulation of CXCR4 (dark zone homing) and CXCR5 (light zone homing) chemokine receptors^54^. Changes in the surface expression of these chemokine receptors occur within several hours, as 10% of GC B cells that migrate from the dark zone to the light zone return to the dark zone within 6 hours^52,54^.

### Tfh dynamics

Tfh and GC-Tfh cells continuously migrate between GCs in lymphoid tissues^55^. Tfh and GC-Tfh cells were recently revealed to migrate into neighboring GCs, unlike GC B cells, which are mostly confined to a single GC in the context of acute protein immunization^55^. Moreover, recently developed Tfh and GC-Tfh cells can participate in ongoing GC reactions, extending the duration of GC responses^55^. This migratory behavior is strictly governed by the regulation of various chemokine receptors and adhesion molecules, including CCR7, CXCR5, PSGL-1, and EBI2, as well as surface receptors such as S1PR2 and PD-1^8,56–59^.

Tfh cells migrate toward CXCL13-rich B-cell follicles by inducing the surface expression of CXCR5 and downregulating the expression of CCR7 and PSGL-1, the membrane proteins required for T-cell homing and retention in the T-cell zone^56,60^. While EBI2, a G protein-coupled receptor activated by 7α,25-dihydroxycholesterol, is required for Tfh cell positioning at the B:T border, it is downregulated when Tfh cells migrate deep into the follicles to induce GCs^61^. The dramatic change in EBI2 expression is controlled partly by Bcl6. Bcl6 also induces CXCR5 expression by inhibiting Blimp1 and Id2, which suppress CXCR5 expression^14,15,62^. Bcl6 simultaneously represses the expression of CCR7, PSGL-1, and EBI2^14,58^, leading Tfh cells to migrate from the T-cell zone to the B:T border and subsequently into B-cell follicles. In addition to Bcl6, Ascl2, Tox2, and STAT1 have been identified as important regulators of these chemokine receptors. Ascl2 acts as a transcriptional activator of the *Cxcr4* and *Cxcr5* genes and as a repressor of the *Ccr7* and *Selplg* genes, respectively^19^. Tox2 directly enhances *Cxcr5* gene expression by augmenting chromatin accessibility^63^, while STAT1 upregulates CXCR5 expression via p300 recruitment^64^. Conversely, Bach2 serves as a transcriptional repressor by binding directly to a regulatory region of the murine *Cxcr5* gene promoter^38^.

Within B-cell follicles, GC-Tfh cells upregulate S1PR2 and PD-1 to adequately support cognate GC B cells by maintaining their localization. This process is partially mediated by S1PR2. The retention of S1PR2-deficient GC-Tfh cells within GCs is reduced without disrupting Tfh differentiation^57^. Furthermore, PD-1 plays an additional role in regulating Tfh positioning by limiting the upregulation of CXCR3. Considering that CXCR3 ligands (CXCL9 and CXCL10) are abundantly expressed in the interfollicular areas outside the follicles^65^, the differential surface expression of PD-1 between Tfh and GC-Tfh cells could determine whether the corresponding cells effectively accumulate inside the GC territory or migrate from the GCs^66^. Although how *S1pr2* gene expression is regulated in Tfh cells remains to be elucidated, both Tox2 and STAT1 have been revealed to induce *Pdcd1* gene expression^63,64^ and therefore might be involved in regulating Tfh and GC-Tfh localization and positioning inside GCs.

Inside GCs, strong CXCR5 expression enables GC-Tfh cells to localize in the light zone, where they place selection pressure on GC-B cells^2^. Interestingly, a fraction of human and mouse GC-Tfh cells were also revealed to express CXCR4^54,67^, suggesting that CXCR4 and CXCR5 potentially cooperate in GC-Tfh positioning, similar to the cyclic migration of GC B cells between the dark zone and the light zone. Indeed, GC-Tfh cells expressing both CXCR4 and CXCR5 preferentially localize to the dark zone^68^. Moreover, experiments using CD4 T-cell-specific *Cxcr4-* or *Cxcr5*-knockout mice revealed that CXCR4-deficient CD4 T cells preferentially localize to the light zone, while CXCR5 deficiency leads to the preferential positioning of GC-Tfh cells to the dark zone^68^. Thus, it is crucial to identify the transcriptional regulation circuits controlling the surface expression of CXCR4 and CXCR5 on GC-Tfh cells to further elucidate the mechanisms of GC-Tfh cells positioning in TD humoral immune responses^68,69^.

## Synapse formation between Tfh cells and B cells

Intravital imaging studies revealed that cells dynamically move across the compartmentalized areas of secondary lymphoid tissues. Inside GCs, Ag-specific GC B cells migrate between the two zones, repeatedly undergoing cell division-somatic hypermutation-positive selection cycles. At the same time, numerous nonspecific B cells migrate in and out of GCs. Thus, the pivotal step occurs when GC B cells, which have successfully evolved their BCR affinity, need to be adequately recognized by GC-Tfh cells in the light zone.

Identification of the correct binding partners is determined via a cell-to-cell interaction-based scanning process of GC-Tfh cells. These cells form immune synapses with B cells (hereafter referred to as B:T synapses) through the engagement of the TCR/MHC-peptide Ag complex, costimulatory molecules such as CD40 ligand (CD40L)/CD40 and ICOS/ICOS-L, and adhesion molecules such as LFA-1/ICAM-1 and SLAMs^70^. In fact, the B:T synapse is crucial for the full maturation of Tfh cells into GC-Tfh cells at the B:T border^71,72^. Thus, the precise control of molecules participating in B:T synapse formation is essential to induce GC formation in B cells in an Ag-specific manner and to guarantee the qualitative evolution of their Ag-binding strength.

Following TCR and CD28 stimulation, the early growth response (Egr) family of transcription factors, including NFAT and possibly Egr-1, binds to the 3’ region of the TATA-proximal NFAT site of the *Cd40lg* gene, inducing its transcription^73^. In contrast, PU.1, an ETS family transcription factor, negatively regulates *Cd40lg* expression by directly binding to the promoter region of the gene. PU.1 deletion in CD4 T cells thus led to a significant increase in Tfh differentiation, enhancing GC formation and TD humoral immunity^74^. TCR- and CD28-mediated signals also trigger AP-1 activation. The AP-1 transcription factor binds to an AP-1 responsive site within the *Icos* promoter, leading to the upregulation of ICOS expression in activated CD4 T cells^75^. The LEF-1 and TCF-1 transcription factors also contribute to the positive regulation of *Icos* expression, ultimately enhancing Tfh differentiation^20^. TCF-1-mediated *Icos* activation can also be induced by Bcl6 via transcriptional repression of Runx2, Runx3, and Klf2, which antagonize ICOS expression in CD4 T cells during Tfh differentiation^14^.

The expression of CD11A, the α subunit of LFA-1, can be regulated by epigenetic modifications. DNA hypomethylation and hyperacetylation of histone H3 at the *CD11A* promoter can be induced by reduced expression of regulatory factor X-box 1 (RFX1), which functions via interaction with DNA methyltransferase 1 (DNMT1) and histone deacetylase 1 (HDAC1) and consequently results in augmented expression of CD11A on CD4 T cells^76^. Additionally, CD11A expression can be enhanced by reduced methylation in the promoter region of the *CD11A* gene by the histone demethylase Jumonji domain-containing protein D3 (JMJD3)^77^.

SAP (SLAM-associated protein), a small signaling adapter protein that functions downstream of SLAM family receptors, facilitates the ability of CD4 T cells to form immune synapses with cognate B cells^78^. SAP-deficient CD4 T cells fail to form stable synapses with cognate B cells and undergo GC-Tfh differentiation; thus, they are not able to support GC formation or induce long-lived plasma cells (LLPCs) or memory B cells^78–81^. Given the critical roles of SAP in Tfh and GC-Tfh cell differentiation and TD humoral immunity, it is very important to understand the transcriptional regulation of the *SH2D1A* (*Sh2d1a*) gene, which encodes SAP. Ets-1 and Ets-2 bind to the Ets consensus site within the *SH2D1A* promoter region. Dominant-negative mutants containing only the DNA binding domain led to a significant decrease in the basal promoter activity of *SH2D1A*, indicating the promotive roles of Ets-1 and Ets-2 in SAP expression^82^. BCL6 is a positive regulator of SAP expression in human CD4 T cells. Ectopic BCL6 expression in tonsillar non-Tfh (CXCR5^-^) and Tfh (CXCR5^int^) cells results in substantial upregulation of SAP expression^25^.

Recently, we revealed a novel mechanism illustrating how CD4 T cells negatively regulate SAP expression to modulate B:T synapse-dependent TD humoral immunity. Mef2d, a member of the myocyte enhancer factor-2 transcription factor family known for its negative regulatory function in neuronal synapse development^83^, plays a similar inhibitory role within the immune system. By binding to a putative Mef2 binding sequence within the murine *Sh2d1a* gene, Mef2d directly suppresses SAP expression in CD4 T cells, inhibiting SAP-dependent B:T synapse formation and TD humoral immune responses^84^.

## Tfh dynamics and B:T synapse formation in vaccines and diseases

B cell help signals are provided by costimulatory molecules and cytokines. CD40L, expressed on Tfh and GC-Tfh cells, triggers the NF-κB pathway downstream of CD40 in the interacting B cells, leading to B-cell proliferation inside GCs^6,85,86^. The interaction between CD40L and CD40 also contributes to a temporal increase in SLAM and CD40 expression on a subset of GC B cells in the light zone, which contributes to further activation of CD40-mediated signals and prolonged interaction with GC-Tfh cells, supporting the division of GC B cells in the dark zone while inhibiting plasma cell differentiation^87^. ICOS-L is upregulated on GC B cells by CD40-mediated activation, which increases the surface area available for engagement with ICOS^hi^ GC-Tfh cells, reinforcing Tfh integrity^71^.

Inside GCs, GC-Tfh cells are an important cellular source of cytokines^88,89^, which are required for proliferation, isotype switching, and antibody-secreting plasma cell differentiation of GC B cells. IL-21 enhances Bcl6 expression in GC B cells and supports GC maintenance, leading to maximal affinity maturation in Ag-specific GC B cells^8,88,90^. This process is supported by IL-4, which exerts antiapoptotic effects on GC B cells that are highly susceptible to apoptosis due partly to genomic instability or nonfunctional BCR expression after somatic hypermutation^3^. These cytokines are also important for the generation of plasma cells^2,89^. IL-21 induces early plasma cell differentiation in IRF4^hi^Bcl6^lo^ GC B cells via STAT3-mediated Blimp1 induction^6,87,91^. With CD40-mediated signals, IL-21 also promotes the positive selection of GC B cells in the light zone^92^. IL-21 and IL-4, together with IFN-γ and IL-17, play important roles in enabling GC B cells to switch their heavy chain constant regions^3,93,94^.

The B-cell help functions of Tfh cells rely on the proper localization of both Tfh and GC-Tfh cells, along with stable cell-to-cell interactions with (GC) B cells. The precise spatiotemporal regulation of the positioning of CD4 T cells and interaction with (GC) B cells is crucial for orchestrating TD humoral immunity. Errors in these regulatory circuits are expected to contribute to poor GC formation or antibody production against exogenous Ags or to elicit uncontrolled GC formation and antibody responses to harmless substances or self-antigens.

EBI2-dependent chemotaxis toward the outer T-cell zone and to the B:T border is important for CD4 T cells to interact with ICOS-L^hi^CD25^+^ DCs, the potent inducers of Tfh differentiation during the priming stage, as well as to make contact with cognate B cells^61^. *Ebi2* deficiency thus severely impairs Tfh differentiation and decreases GC formation and plasma cell differentiation after acute bacterial infection or immunization with SRBCs^61^. After GCs form, GC-Tfh cells downregulate EBI2 expression for GC retention rather than migrating toward the T-cell zone^58^. At the same time, GC-Tfh cells induce the expression of S1PR2, which binds to sphingosine 1-phosphate (S1P), the ligand chemoattracting B cells toward the inner follicle. Although the lack of the *S1pr2* gene encoding S1PR2 is insufficient to impede GC-Tfh retention inside GCs, S1PR2 appears to play redundant roles with CXCR5 in controlling the positioning of GC-Tfh cells, enabling interactions with GC B cells in GCs^57^.

Positive selection of GC B cells occurs in the light zone of GCs^2,6^. Thus, the mislocalization of GC-Tfh cells outside the light zone could have profound impacts on the quantity or quality of Ag-specific B-cell responses. This process is negatively controlled partly by the CXCR4 chemokine receptor^68^. CXCR4 expression in GC-Tfh cells was significantly enhanced in aged mice, which led to the preferential positioning of GC-Tfh cells in the CXCL12-rich dark zone. The mislocalization of GC-Tfh cells in the dark zone led to reduced cellular outputs of GCs in aged mice after immunization, which was restored by adoptive transfer of young CD4 T cells^68^.

As explained above, positive selection guarantees that Ag-specific B cells clonally expand and undergo affinity maturation. Tfh and GC-Tfh cells scan B cells and GC B cells whether they present appropriate peptide Ags and form stable interactions. Molecules contributing to the stable interaction between Tfh cells and B cells at the B:T border and between GC-Tfh cells and GC B cells in the light zone thus function as limiting factors for B cells to form GC responses. Genetic ablation of ICOS and CD40L^95,96^ or blocking antibodies interfering with the CD40-CD40L or ICOS-ICOS-L interaction^97,98^ results in poor GC formation and impaired production of isotype-switched affinity-matured Abs. In contrast, enhanced ICOS expression in CD4 T cells or *CD40LG* duplication is strongly associated with the unchecked induction of autoantibodies in autoimmune disorders such as systemic lupus erythematosus and rheumatoid arthritis^99–101^. Moreover, failures in the epigenetic regulation of CD11A expression (discussed above) are associated with enhanced Tfh differentiation in SLE patients^76^, fueling T and B-cell hyperactivity and exacerbating SLE pathogenesis^77^.

Among the molecules functioning in the B:T synapse, SAP is very important. CD4 T cells require SAP expression to interact with B cells but not with non-B-cell APCs^78^. Inside GCs, the presence of SAP determines whether GC-Tfh cells make sufficient contact with B cells to provide positive selection signals in the light zone. Loss-of-function mutations in the *SH2D1A* gene contribute to the development of type 1 X-linked lymphoproliferative (XLP) syndrome^102^, leading patients to suffer from clinical manifestations, such as hypogammaglobulinemia and recurrent bacterial or viral infections. Thus, transcriptional regulation of SAP expression in CD4 T cells might control TD humoral immunity. Intriguingly, we recently found that CD4 T-cell-specific deletion of Mef2d led to a significant increase in Ag-specific GC formation and antibody production after immunization, while a profound reduction in *MEF2D* mRNA expression in CD4 T cells was strongly correlated with various autoimmune parameters in patients with SLE^84^.

**Fig. 1:**
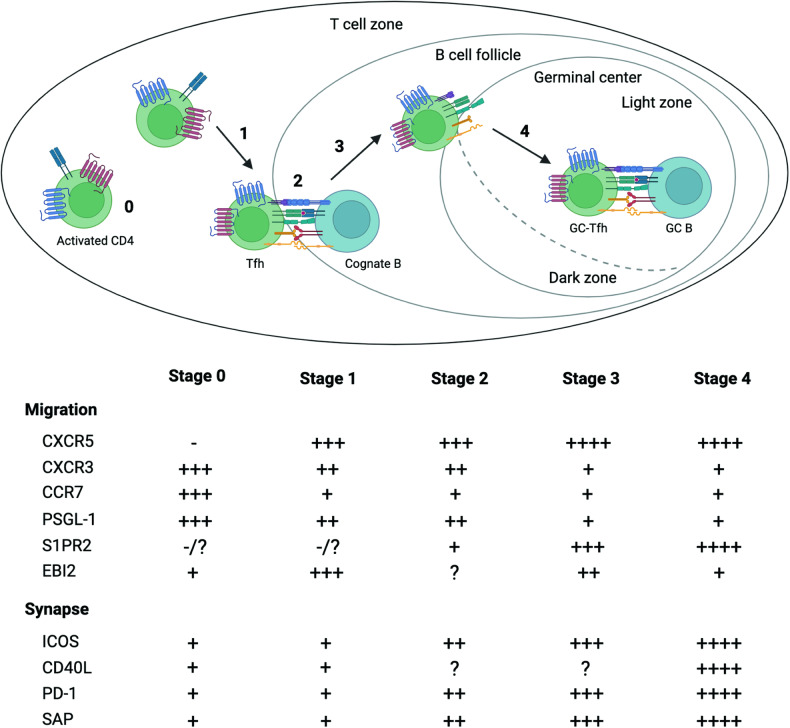
Tfh dynamics and synapse formation with B cells and GC B cells. **Stage 0:** Naive CD4 T cells are activated in the T-cell zone of lymphoid tissues after recognition of antigens presented by DCs. These cells express high levels of receptors for chemokines and chemoattractant molecules present in the T-cell zone. **Stage 1:** Pre-Tfh cells, which are formed during interaction with DCs, upregulate CXCR5 while downregulating CCR7 and PSGL-1 and migrate to the B:T border. **Stage 2:** At the B:T border, pre-Tfh cells interact with cognate B cells. This interaction is supported by ICOS, CD40L, and SAP, which drive full maturation of Tfh cells into GC-Tfh cells. **Stage 3:** To ensure further migration into GC areas, these cells increase CXCR5 and PD-1 expression while decreasing the expression of chemoattractant receptors, including EBI2 and PSGL-1. **Stage 4:** Inside GCs, GC-Tfh cells upregulate CXCR5, S1PR2, and PD-1 to maintain their localization in the light zone of the GC and upregulate synapse-related molecules to stably interact with GC B cells that present cognate peptide Ags.

**Fig. 2:**
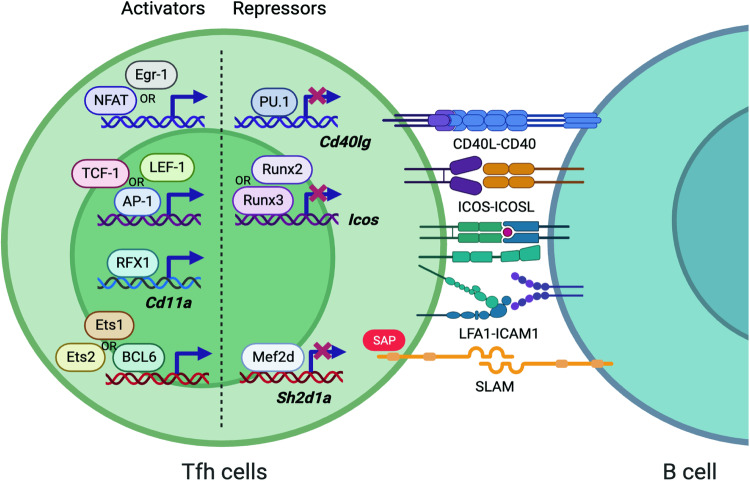
Transcriptional regulation of immune synapse formation molecules by Tfh cells. The transcriptional regulatory network controls the expression of molecules involved in immune synapse formation between Tfh and B cells and between GC-Tfh cells and GC B cells. **Activators (Egr-1, TCF-1, RFX1, and BCL6):** upregulate the expression of the *Cd40lg*, *Icos*, *Cd11a*, and *Sh2d1a* genes, respectively. **Repressors (PU.1, Runx3, and Mef2d):** downregulate the expression of *Cd40lg*, *Icos*, and *Sh2d1a*. The positive and negative regulators of these genes highlight that the B:T immune synapse requires an intricate genetic program to properly elicit powerful TD humoral immune responses.

A comprehensive understanding of the transcriptional regulation of Tfh cells, particularly its impact on Tfh dynamics (Fig. 1) and synapse formation with B cells (Fig. 2), would provide insights for the development of innovative therapeutic strategies for the treatment of infectious diseases and autoimmune disorders.

## References

[1] Crotty, S. T follicular helper cell differentiation, function, and roles in disease. *Immunity* **41**, 529–542 (2014). 25367570 10.1016/j.immuni.2014.10.004 PMC4223692

[2] Victora, G. D. & Nussenzweig, M. C. Germinal centers. *Annu. Rev. Immunol.* **40**, 413–442 (2022). 35113731 10.1146/annurev-immunol-120419-022408

[3] Crotty, S. Follicular helper CD4 T cells (TFH). *Annu. Rev. Immunol.* **29**, 621–663 (2011). 21314428 10.1146/annurev-immunol-031210-101400

[4] Choi, J. & Crotty, S. Bcl6-mediated transcriptional regulation of follicular helper T cells (T(FH)). *Trends Immunol.* **42**, 336–349 (2021). 33663954 10.1016/j.it.2021.02.002 PMC8021443

[5] Choi, Y. S. et al. ICOS receptor instructs T follicular helper cell versus effector cell differentiation via induction of the transcriptional repressor Bcl6. *Immunity* **34**, 932–946 (2011). 21636296 10.1016/j.immuni.2011.03.023 PMC3124577

[6] Crotty, S. T Follicular helper cell biology: a decade of discovery and diseases. *Immunity* **50**, 1132–1148 (2019). 31117010 10.1016/j.immuni.2019.04.011 PMC6532429

[7] Victora, G. D. & Nussenzweig, M. C. Germinal centers. *Annu. Rev. Immunol.* **30**, 429–457 (2012). 22224772 10.1146/annurev-immunol-020711-075032

[8] Vinuesa, C. G., Linterman, M. A., Yu, D. & MacLennan, I. C. Follicular helper T cells. *Annu. Rev. Immunol.* **34**, 335–368 (2016). 26907215 10.1146/annurev-immunol-041015-055605

[9] DiToro, D. et al. Differential IL-2 expression defines developmental fates of follicular versus nonfollicular helper T cells. *Science* **361**, eaao2933 (2018). 30213884 10.1126/science.aao2933 PMC6501592

[10] Tubo, N. J. et al. Single naive CD4+ T cells from a diverse repertoire produce different effector cell types during infection. *Cell* **153**, 785–796 (2013). 23663778 10.1016/j.cell.2013.04.007 PMC3766899

[11] Gigoux, M. et al. Inducible costimulator promotes helper T-cell differentiation through phosphoinositide 3-kinase. *Proc. Natl Acad. Sci. USA* **106**, 20371–20376 (2009). 19915142 10.1073/pnas.0911573106 PMC2787139

[12] Pedros, C. et al. A TRAF-like motif of the inducible costimulator ICOS controls development of germinal center TFH cells via the kinase TBK1. *Nat. Immunol.* **17**, 825–833 (2016). 27135603 10.1038/ni.3463 PMC4915981

[13] Weber, J. P. et al. ICOS maintains the T follicular helper cell phenotype by down-regulating Kruppel-like factor 2. *J. Exp. Med.* **212**, 217–233 (2015). 25646266 10.1084/jem.20141432 PMC4322049

[14] Choi, J. et al. Bcl-6 is the nexus transcription factor of T follicular helper cells via repressor-of-repressor circuits. *Nat. Immunol.* **21**, 777–789 (2020). 32572238 10.1038/s41590-020-0706-5 PMC7449381

[15] Hatzi, K. et al. BCL6 orchestrates Tfh cell differentiation via multiple distinct mechanisms. *J. Exp. Med.* **212**, 539–553 (2015). 25824819 10.1084/jem.20141380 PMC4387288

[16] Johnston, R. J. et al. Bcl6 and Blimp-1 are reciprocal and antagonistic regulators of T follicular helper cell differentiation. *Science* **325**, 1006–1010 (2009). 19608860 10.1126/science.1175870 PMC2766560

[17] Nurieva, R. I. et al. Bcl6 mediates the development of T follicular helper cells. *Science* **325**, 1001–1005 (2009). 19628815 10.1126/science.1176676 PMC2857334

[18] Yu, D. et al. The transcriptional repressor Bcl-6 directs T follicular helper cell lineage commitment. *Immunity* **31**, 457–468 (2009). 19631565 10.1016/j.immuni.2009.07.002

[19] Liu, X. et al. Transcription factor achaete-scute homologue 2 initiates follicular T-helper-cell development. *Nature* **507**, 513–518 (2014). 24463518 10.1038/nature12910 PMC4012617

[20] Choi, Y. S. et al. LEF-1 and TCF-1 orchestrate T(FH) differentiation by regulating differentiation circuits upstream of the transcriptional repressor Bcl6. *Nat. Immunol.* **16**, 980–990 (2015). 26214741 10.1038/ni.3226 PMC4545301

[21] Wu, T. et al. TCF1 is required for the T follicular helper cell response to viral infection. *Cell Rep.* **12**, 2099–2110 (2015). 26365183 10.1016/j.celrep.2015.08.049 PMC4591235

[22] Xu, L. et al. The transcription factor TCF-1 initiates the differentiation of T(FH) cells during acute viral infection. *Nat. Immunol.* **16**, 991–999 (2015). 26214740 10.1038/ni.3229

[23] Shao, P. et al. Cutting edge: Tcf1 instructs T follicular helper cell differentiation by repressing Blimp1 in response to acute viral infection. *J. Immunol.* **203**, 801–806 (2019). 31300510 10.4049/jimmunol.1900581 PMC6684471

[24] Andris, F. et al. The transcription factor c-Maf promotes the differentiation of follicular helper T cells. *Front. Immunol.* **8**, 480 (2017). 28496444 10.3389/fimmu.2017.00480 PMC5406410

[25] Kroenke, M. A. et al. Bcl6 and Maf cooperate to instruct human follicular helper CD4 T cell differentiation. *J. Immunol.* **188**, 3734–3744 (2012). 22427637 10.4049/jimmunol.1103246 PMC3324673

[26] Zhu, F. et al. Spatiotemporal resolution of germinal center Tfh cell differentiation and divergence from central memory CD4(+) T cell fate. *Nat. Commun.* **14**, 3611 (2023). 37330549 10.1038/s41467-023-39299-3 PMC10276816

[27] Ise, W. et al. The transcription factor BATF controls the global regulators of class-switch recombination in both B cells and T cells. *Nat. Immunol.* **12**, 536–543 (2011). 21572431 10.1038/ni.2037 PMC3117275

[28] Bollig, N. et al. Transcription factor IRF4 determines germinal center formation through follicular T-helper cell differentiation. *Proc. Natl Acad. Sci. USA* **109**, 8664–8669 (2012). 22552227 10.1073/pnas.1205834109 PMC3365194

[29] Kwon, H. et al. Analysis of interleukin-21-induced Prdm1 gene regulation reveals functional cooperation of STAT3 and IRF4 transcription factors. *Immunity* **31**, 941–952 (2009). 20064451 10.1016/j.immuni.2009.10.008 PMC3272079

[30] Hiramatsu, Y. et al. c-Maf activates the promoter and enhancer of the IL-21 gene, and TGF-beta inhibits c-Maf-induced IL-21 production in CD4+ T cells. *J. Leukoc. Biol.* **87**, 703–712 (2010). 20042469 10.1189/jlb.0909639

[31] Eto, D. et al. IL-21 and IL-6 are critical for different aspects of B cell immunity and redundantly induce optimal follicular helper CD4 T cell (Tfh) differentiation. *PLoS ONE* **6**, e17739 (2011). 21423809 10.1371/journal.pone.0017739 PMC3056724

[32] Choi, Y. S., Eto, D., Yang, J. A., Lao, C. & Crotty, S. Cutting edge: STAT1 is required for IL-6-mediated Bcl6 induction for early follicular helper cell differentiation. *J. Immunol.* **190**, 3049–3053 (2013). 23447690 10.4049/jimmunol.1203032 PMC3626564

[33] Papillion, A. et al. Inhibition of IL-2 responsiveness by IL-6 is required for the generation of GC-T(FH) cells. *Sci. Immunol.* **4**, eaaw7636 (2019). 31519812 10.1126/sciimmunol.aaw7636 PMC6820141

[34] Schmitt, N. et al. The cytokine TGF-beta co-opts signaling via STAT3-STAT4 to promote the differentiation of human TFH cells. *Nat. Immunol.* **15**, 856–865 (2014). 25064073 10.1038/ni.2947 PMC4183221

[35] Basso, K. & Dalla-Favera, R. Germinal centres and B cell lymphomagenesis. *Nat. Rev. Immunol.* **15**, 172–184 (2015). 25712152 10.1038/nri3814

[36] Shaffer, A. L. et al. Blimp-1 orchestrates plasma cell differentiation by extinguishing the mature B cell gene expression program. *Immunity* **17**, 51–62 (2002). 12150891 10.1016/s1074-7613(02)00335-7

[37] Tunyaplin, C. et al. Direct repression of prdm1 by Bcl-6 inhibits plasmacytic differentiation. *J. Immunol.* **173**, 1158–1165 (2004). 15240705 10.4049/jimmunol.173.2.1158

[38] Geng, J. et al. Bach2 negatively regulates T follicular helper cell differentiation and is critical for CD4(+) T cell memory. *J. Immunol.* **202**, 2991–2998 (2019). 30971440 10.4049/jimmunol.1801626 PMC6504585

[39] Lahmann, A. et al. Bach2 controls T follicular helper cells by direct repression of Bcl-6. *J. Immunol.* **202**, 2229–2239 (2019). 30833348 10.4049/jimmunol.1801400

[40] Stone, E. L. et al. ICOS coreceptor signaling inactivates the transcription factor FOXO1 to promote Tfh cell differentiation. *Immunity* **42**, 239–251 (2015). 25692700 10.1016/j.immuni.2015.01.017 PMC4334393

[41] Wang, H. et al. The transcription factor Foxp1 is a critical negative regulator of the differentiation of follicular helper T cells. *Nat. Immunol.* **15**, 667–675 (2014). 24859450 10.1038/ni.2890 PMC4142638

[42] Smith, K. A. Interleukin-2: inception, impact, and implications. *Science* **240**, 1169–1176 (1988). 3131876 10.1126/science.3131876

[43] Ballesteros-Tato, A. et al. Interleukin-2 inhibits germinal center formation by limiting T follicular helper cell differentiation. *Immunity* **36**, 847–856 (2012). 22464171 10.1016/j.immuni.2012.02.012 PMC3361521

[44] Johnston, R. J., Choi, Y. S., Diamond, J. A., Yang, J. A. & Crotty, S. STAT5 is a potent negative regulator of TFH cell differentiation. *J. Exp. Med.* **209**, 243–250 (2012). 22271576 10.1084/jem.20111174 PMC3281266

[45] Locci, M. et al. Activin A programs the differentiation of human TFH cells. *Nat. Immunol.* **17**, 976–984 (2016). 27376469 10.1038/ni.3494 PMC4955732

[46] McDonald, P. W. et al. IL-7 signalling represses Bcl-6 and the TFH gene program. *Nat. Commun.* **7**, 10285 (2016). 26743592 10.1038/ncomms10285 PMC4729877

[47] Baessler, A. et al. Tet2 coordinates with Foxo1 and Runx1 to balance T follicular helper cell and T helper 1 cell differentiation. *Sci. Adv.* **8**, eabm4982 (2022). 35704571 10.1126/sciadv.abm4982 PMC9200277

[48] Li, F. et al. Ezh2 programs T(FH) differentiation by integrating phosphorylation-dependent activation of Bcl6 and polycomb-dependent repression of p19Arf. *Nat. Commun.* **9**, 5452 (2018). 30575739 10.1038/s41467-018-07853-z PMC6303346

[49] Belanger, S. et al. The chromatin regulator Mll1 supports T follicular helper cell differentiation by controlling expression of Bcl6, LEF-1, and TCF-1. *J. Immunol.* **210**, 1752–1760 (2023). 37074193 10.4049/jimmunol.2200927 PMC10334568

[50] Bannard, O. et al. Germinal center centroblasts transition to a centrocyte phenotype according to a timed program and depend on the dark zone for effective selection. *Immunity* **39**, 912–924 (2013). 24184055 10.1016/j.immuni.2013.08.038 PMC3828484

[51] Cyster, J. G. & Allen, C. D. C. B cell responses: cell interaction dynamics and decisions. *Cell* **177**, 524–540 (2019). 31002794 10.1016/j.cell.2019.03.016 PMC6538279

[52] Victora, G. D. et al. Germinal center dynamics revealed by multiphoton microscopy with a photoactivatable fluorescent reporter. *Cell* **143**, 592–605 (2010). 21074050 10.1016/j.cell.2010.10.032 PMC3035939

[53] Muppidi, J. R. et al. Loss of signalling via Galpha13 in germinal centre B-cell-derived lymphoma. *Nature* **516**, 254–258 (2014). 25274307 10.1038/nature13765 PMC4267955

[54] Allen, C. D. et al. Germinal center dark and light zone organization is mediated by CXCR4 and CXCR5. *Nat. Immunol.* **5**, 943–952 (2004). 15300245 10.1038/ni1100

[55] Shulman, Z. et al. T follicular helper cell dynamics in germinal centers. *Science* **341**, 673–677 (2013). 23887872 10.1126/science.1241680 PMC3941467

[56] Haynes, N. M. et al. Role of CXCR5 and CCR7 in follicular Th cell positioning and appearance of a programmed cell death gene-1high germinal center-associated subpopulation. *J. Immunol.* **179**, 5099–5108 (2007). 17911595 10.4049/jimmunol.179.8.5099

[57] Moriyama, S. et al. Sphingosine-1-phosphate receptor 2 is critical for follicular helper T cell retention in germinal centers. *J. Exp. Med.* **211**, 1297–1305 (2014). 24913235 10.1084/jem.20131666 PMC4076581

[58] Suan, D. et al. T follicular helper cells have distinct modes of migration and molecular signatures in naive and memory immune responses. *Immunity* **42**, 704–718 (2015). 25840682 10.1016/j.immuni.2015.03.002

[59] Yeh, C. H., Finney, J., Okada, T., Kurosaki, T. & Kelsoe, G. Primary germinal center-resident T follicular helper cells are a physiologically distinct subset of CXCR5(hi)PD-1(hi) T follicular helper cells. *Immunity* **55**, 272–289.e7 (2022). 10.1016/j.immuni.2021.12.015 PMC8842852 35081372

[60] Poholek, A. C. et al. In vivo regulation of Bcl6 and T follicular helper cell development. *J. Immunol.* **185**, 313–326 (2010). 20519643 10.4049/jimmunol.0904023 PMC2891136

[61] Li, J., Lu, E., Yi, T. & Cyster, J. G. EBI2 augments Tfh cell fate by promoting interaction with IL-2-quenching dendritic cells. *Nature* **533**, 110–114 (2016). 27147029 10.1038/nature17947 PMC4883664

[62] Shaw, L. A. et al. Id2 reinforces TH1 differentiation and inhibits E2A to repress TFH differentiation. *Nat. Immunol.* **17**, 834–843 (2016). 27213691 10.1038/ni.3461 PMC4915968

[63] Xu, W. et al. The transcription factor Tox2 drives T follicular helper cell development via regulating chromatin accessibility. *Immunity* **51**, 826–839.e5 (2019). 31732165 10.1016/j.immuni.2019.10.006

[64] Nakayamada, S. et al. Type I IFN induces binding of STAT1 to Bcl6: divergent roles of STAT family transcription factors in the T follicular helper cell genetic program. *J. Immunol.* **192**, 2156–2166 (2014). 24489092 10.4049/jimmunol.1300675 PMC3967131

[65] Groom, J. R. et al. CXCR3 chemokine receptor-ligand interactions in the lymph node optimize CD4+ T helper 1 cell differentiation. *Immunity* **37**, 1091–1103 (2012). 23123063 10.1016/j.immuni.2012.08.016 PMC3525757

[66] Shi, J. et al. PD-1 controls follicular T helper cell positioning and function. *Immunity* **49**, 264–274.e4 (2018). 30076099 10.1016/j.immuni.2018.06.012 PMC6104813

[67] Elsner, R. A., Ernst, D. N. & Baumgarth, N. Single and coexpression of CXCR4 and CXCR5 identifies CD4 T helper cells in distinct lymph node niches during influenza virus infection. *J. Virol.* **86**, 7146–7157 (2012). 22532671 10.1128/JVI.06904-11 PMC3416343

[68] Silva-Cayetano, A. et al. Spatial dysregulation of T follicular helper cells impairs vaccine responses in aging. *Nat. Immunol.* **24**, 1124–1137 (2023). 37217705 10.1038/s41590-023-01519-9 PMC10307630

[69] Mondal, A., Sawant, D. & Dent, A. L. Transcriptional repressor BCL6 controls Th17 responses by controlling gene expression in both T cells and macrophages. *J. Immunol.* **184**, 4123–4132 (2010). 20212093 10.4049/jimmunol.0901242

[70] Papa, I. & Vinuesa, C. G. Synaptic interactions in germinal centers. *Front. Immunol.* **9**, 1858 (2018). 30150988 10.3389/fimmu.2018.01858 PMC6099157

[71] Liu, D. et al. T-B-cell entanglement and ICOSL-driven feed-forward regulation of germinal centre reaction. *Nature* **517**, 214–218 (2015). 25317561 10.1038/nature13803

[72] Zaretsky, I. et al. ICAMs support B cell interactions with T follicular helper cells and promote clonal selection. *J. Exp. Med.* **214**, 3435–3448 (2017). 28939548 10.1084/jem.20171129 PMC5679169

[73] Lindgren, H., Axcrona, K. & Leanderson, T. Regulation of transcriptional activity of the murine CD40 ligand promoter in response to signals through TCR and the costimulatory molecules CD28 and CD2. *J. Immunol.* **166**, 4578–4585 (2001). 11254715 10.4049/jimmunol.166.7.4578

[74] Awe, O. et al. PU.1 expression in T follicular helper cells limits CD40L-dependent germinal center B cell development. *J. Immunol.* **195**, 3705–3715 (2015). 26363052 10.4049/jimmunol.1500780 PMC4592843

[75] Watanabe, M. et al. AP-1 is involved in ICOS gene expression downstream of TCR/CD28 and cytokine receptor signaling. *Eur. J. Immunol.* **42**, 1850–1862 (2012). 22585681 10.1002/eji.201141897

[76] Zhao, M. et al. RFX1 regulates CD70 and CD11a expression in lupus T cells by recruiting the histone methyltransferase SUV39H1. *Arthritis Res. Ther.* **12**, R227 (2010). 21192791 10.1186/ar3214 PMC3046540

[77] Yin, H. et al. Histone demethylase JMJD3 regulates CD11a expression through changes in histone H3K27 tri-methylation levels in CD4+ T cells of patients with systemic lupus erythematosus. *Oncotarget* **8**, 48938–48947 (2017). 28430662 10.18632/oncotarget.16894 PMC5564738

[78] Qi, H., Cannons, J. L., Klauschen, F., Schwartzberg, P. L. & Germain, R. N. SAP-controlled T-B cell interactions underlie germinal centre formation. *Nature* **455**, 764–769 (2008). 18843362 10.1038/nature07345 PMC2652134

[79] Cannons, J. L. et al. SAP regulates T cell-mediated help for humoral immunity by a mechanism distinct from cytokine regulation. *J. Exp. Med.* **203**, 1551–1565 (2006). 16754717 10.1084/jem.20052097 PMC2118305

[80] Crotty, S., Kersh, E. N., Cannons, J., Schwartzberg, P. L. & Ahmed, R. SAP is required for generating long-term humoral immunity. *Nature* **421**, 282–287 (2003). 12529646 10.1038/nature01318

[81] Veillette, A. et al. SAP expression in T cells, not in B cells, is required for humoral immunity. *Proc. Natl Acad. Sci. USA* **105**, 1273–1278 (2008). 18212118 10.1073/pnas.0710698105 PMC2234128

[82] Okamoto, S. et al. Expression of the SH2D1A gene is regulated by a combination of transcriptional and post-transcriptional mechanisms. *Eur. J. Immunol.* **34**, 3176–3186 (2004). 15459902 10.1002/eji.200324755

[83] Flavell, S. W. et al. Activity-dependent regulation of MEF2 transcription factors suppresses excitatory synapse number. *Science* **311**, 1008–1012 (2006). 16484497 10.1126/science.1122511

[84] Kim, Y. J. et al. The transcription factor Mef2d regulates B:T synapse-dependent GC-T(FH) differentiation and IL-21-mediated humoral immunity. *Sci. Immunol.* **8**, eadf2248 (2023). 36961907 10.1126/sciimmunol.adf2248 PMC10311795

[85] Lee, S. K. et al. B cell priming for extrafollicular antibody responses requires Bcl-6 expression by T cells. *J. Exp. Med.* **208**, 1377–1388 (2011). 21708925 10.1084/jem.20102065 PMC3135363

[86] Luo, W., Weisel, F. & Shlomchik, M. J. B cell receptor and CD40 signaling are rewired for synergistic induction of the c-Myc transcription factor in germinal center B cells. *Immunity* **48**, 313–326.e5 (2018). 29396161 10.1016/j.immuni.2018.01.008 PMC5821563

[87] Ise, W. et al. T Follicular helper cell-germinal center B cell interaction strength regulates entry into plasma cell or recycling germinal center cell fate. *Immunity* **48**, 702–715.e4 (2018). 29669250 10.1016/j.immuni.2018.03.027

[88] Linterman, M. A. et al. IL-21 acts directly on B cells to regulate Bcl-6 expression and germinal center responses. *J. Exp. Med.* **207**, 353–363 (2010). 20142429 10.1084/jem.20091738 PMC2822609

[89] Weinstein, J. S. et al. TFH cells progressively differentiate to regulate the germinal center response. *Nat. Immunol.* **17**, 1197–1205 (2016). 27573866 10.1038/ni.3554 PMC5030190

[90] Zotos, D. et al. IL-21 regulates germinal center B cell differentiation and proliferation through a B cell-intrinsic mechanism. *J. Exp. Med.* **207**, 365–378 (2010). 20142430 10.1084/jem.20091777 PMC2822601

[91] Krautler, N. J. et al. Differentiation of germinal center B cells into plasma cells is initiated by high-affinity antigen and completed by Tfh cells. *J. Exp. Med.* **214**, 1259–1267 (2017). 28363897 10.1084/jem.20161533 PMC5413338

[92] Luo, W. et al. IL-21R signal reprogramming cooperates with CD40 and BCR signals to select and differentiate germinal center B cells. *Sci. Immunol.* **8**, eadd1823 (2023). 36800413 10.1126/sciimmunol.add1823 PMC10206726

[93] Meli, A. P., Fontes, G., Leung Soo, C. & King, I. L. T Follicular helper cell-derived IL-4 is required for IgE production during intestinal helminth infection. *J. Immunol.* **199**, 244–252 (2017). 28533444 10.4049/jimmunol.1700141

[94] Yang, Z., Wu, C. M., Targ, S. & Allen, C. D. C. IL-21 is a broad negative regulator of IgE class switch recombination in mouse and human B cells. *J. Exp. Med.* **217**, e20190472 (2020). 32130409 10.1084/jem.20190472 PMC7201927

[95] Lougaris, V., Badolato, R., Ferrari, S. & Plebani, A. Hyper immunoglobulin M syndrome due to CD40 deficiency: clinical, molecular, and immunological features. *Immunol. Rev.* **203**, 48–66 (2005). 15661021 10.1111/j.0105-2896.2005.00229.x

[96] Warnatz, K. et al. Human ICOS deficiency abrogates the germinal center reaction and provides a monogenic model for common variable immunodeficiency. *Blood* **107**, 3045–3052 (2006). 16384931 10.1182/blood-2005-07-2955

[97] Cicalese, M. P. et al. Circulating follicular helper and follicular regulatory T cells are severely compromised in human CD40 deficiency: a case report. *Front. Immunol.* **9**, 1761 (2018). 30131802 10.3389/fimmu.2018.01761 PMC6090258

[98] Uwadiae, F. I., Pyle, C. J., Walker, S. A., Lloyd, C. M. & Harker, J. A. Targeting the ICOS/ICOS-L pathway in a mouse model of established allergic asthma disrupts T follicular helper cell responses and ameliorates disease. *Allergy* **74**, 650–662 (2019). 30220084 10.1111/all.13602 PMC6492018

[99] Okamoto, T. et al. Expression and function of the co-stimulator H4/ICOS on activated T cells of patients with rheumatoid arthritis. *J. Rheumatol.* **30**, 1157–1163 (2003). 12784384

[100] Le Coz, C. et al. CD40LG duplication-associated autoimmune disease is silenced by nonrandom X-chromosome inactivation. *J. Allergy Clin. Immunol.* **141**, 2308–2311.e2307 (2018). 29499223 10.1016/j.jaci.2018.02.010 PMC5994181

[101] Yang, J. H. et al. Expression and function of inducible costimulator on peripheral blood T cells in patients with systemic lupus erythematosus. *Rheumatology* **44**, 1245–1254 (2005). 15987711 10.1093/rheumatology/keh724

[102] Pachlopnik, S. J. et al. Clinical similarities and differences of patients with X-linked lymphoproliferative syndrome type 1 (XLP-1/SAP deficiency) versus type 2 (XLP-2/XIAP deficiency). *Blood* **117**, 1522–1529 (2011). 21119115 10.1182/blood-2010-07-298372

